# Accurate and rapid single nucleotide variation detection in *PCSK9* gene using nanopore sequencing

**DOI:** 10.3389/fmed.2025.1620405

**Published:** 2025-08-26

**Authors:** Ilaria Massaiu, Vincenza Valerio, Valentina Rusconi, Francesca Bertolini, Donato De Giorgi, Veronika A. Myasoedova, Paolo Poggio

**Affiliations:** ^1^Centro Cardiologico Monzino IRCCS, Milan, Italy; ^2^Department of Pharmacy, University of Naples “Federico II”, Naples, Italy; ^3^Department of Biomedical, Surgical and Dental Sciences, University of Milan, Milan, Italy

**Keywords:** third-generation sequencing, nanopore sequencing, single nucleotide variation, variant calling, *PCSK9*, cardiovascular disease

## Abstract

**Background:**

Genetic testing is essential for disease screening, diagnosis, prognosis, and pharmacotherapy guidance. Oxford Nanopore Technologies (ONT) offers a cost-effective platform for long-read sequencing, yet its routine use in clinical diagnostics remains under evaluation. We tested different nanopore sequencing pipelines aimed at accurately detecting single-nucleotide variants (SNV) in a gene locus spanning ⁓25 kb.

**Methods:**

As a proof of concept, *PCSK9* was selected for its relevance to cardiovascular disease and suitable sequence structure. Twelve subjects were analyzed using different sequencing flow cells, basecalling models, and SNV calling algorithms. Sanger sequencing served as the reference for performance validation. Sequencing throughput per flow cell was also estimated.

**Results:**

The combination of super high accuracy (SUP) basecalling with Longshot variant calling demonstrated the highest performance across flow cells. MinION flow cell reached a perfect F1-score of 100%, while the more cost-effective Flongle flow cell remained a viable alternative (mean F1-score: 98.2% ± 4.2). Throughput analysis indicated that a single MinION flow cell could process up to 96 samples and ⁓40 long sequencing regions, whereas a Flongle flow cell could support sequencing of 96 samples and one long region.

**Conclusion:**

The proposed nanopore-based SNV identification workflows may support the development of long-read, targeted gene panels, offering a promising tool for both diagnostic and discovery applications, particularly in multi-gene settings such as oncology and cardiology.

## 1 Introduction

The identification and characterization of genetic variants linked to human diseases and disorders are crucial in clinical medicine for an early diagnosis and the discover of new therapeutic targets (1). The development of Next-Generation sequencing (NGS) technologies allowed large-scale and rapid assessment of the entire human genome reducing the cost with respect to the traditional Sanger sequencing method but preserving the accuracy. Short-read sequencing (SRS), the most commonly used form of NGS, has for many years been considered a standard for diagnostic tests due to its ability to produce nearly error-free sequences at very low cost. However, the sequencing limited to short-read fragments (150–300 bp) makes challenging the detection of variants in repetitive and structurally complex regions of the genome (1). In this regard, the advent of long-read sequencing (LRS) methods, namely Pacific Bioscience (PacBio) (2) and Oxford Nanopore technology (ONT) (3), have led to resolve molecular diagnoses where SRS fails (4, 5), specifically for the detection of structural variants (SVs). Nevertheless, the adoption of these LRS methods was initially challenging because of the high sequencing error rate. In recent years, the ONT platform has undergone major improvements, both in its chemical kits and bioinformatics tools, which have led an exponential increase in sequencing accuracy [single-read accuracy > 99% was reached with the latest released kits and tools (6)]. Thereby, ONT approach is gaining popularity for genetic testing (4, 7).

The concept behind ONT, based on a direct measurement of the changes in ionic current when single-stranded nucleic acids pass through biological pores (8), allows real-time analysis of short to ultra-long fragments of DNA/RNA, in fully scalable formats. MinION and Flongle flow cells are sequencing units designed for use with the MinION sequencer, offering flexibility for a wide range of applications. A key factor determining the quality and usability of ONT sequencing is the basecalling, the computational process of translating raw electrical signal to nucleotide sequence. Different basecalling tools, based on neural network, have been proposed from ONT to date, such as Albacore, Guppy, and Dorado. Moreover, the success application of ONT platforms in clinical genomics was determined to its ability to identify single-nucleotide variants (SNVs), short insertions and/or deletions (indel), and SVs. Therefore, numerous deep learning and statistical-based methods tailored for long-read-based variant calling were proposed (9–12).

Despite the innovative and attractive features offered by ONT platform, it is not yet considered the gold standard in clinical diagnostics due to several limitations and challenges that prevent it from meeting the stringent requirements for routine clinical use. In this regard, targeted NGS panels are the routine diagnostic tool of well-characterized familial disorders, such as familial hypercholesterolemia with a panel that integrate key genes implicated in lipid regulation, including proprotein convertase subtilisin/kexin type 9 (*PCSK9*) along with low density lipoprotein receptor (*LDLR*) and apolipoprotein B (*APOB*) genes (13). By focusing on a curated set of clinically relevant genes, gene panels provide a cost-effective, rapid, and highly sensitive method for identifying disease-causing mutations. This targeted approach minimizes the complexity of analysis and interpretation compared to broader techniques, such as whole-exome sequencing (WES) or whole-genome sequencing (WGS), while still maintaining high diagnostic accuracy. However, patients evaluated by specific genetic panels limits the assessment of other possible SNVs in long genes and the discovery of new ones.

Therefore, we aimed to set up and validate a nanopore sequencing workflow capable of detecting SNVs in long region in a timely and cost-effective manner, using the *PCSK9* locus (~25 kb) as an example application.

## 2 Materials and methods

### 2.1 *PCSK9* primers design and polymerase chain reaction (PCR) setup

*PCSK9* gene is located on chromosome 1 and spans 25′406 bp, from 55′039’548 to 55′064’852 (hg19). In order to ensure the complete sequencing of this gene, three different primers were designed using PrimalScheme (14)^1^ and ordered from IDT (Integrated DNA Technologies, Iowa, United States; Supplementary Table S1). Oligos were resuspended at a final concentration of 100 μM using sterile DNAse free water. Working aliquots were created by diluting forward and reverse primers to a final concentration of 10 μM.

Although ONT enables sequencing of ultra-long reads exceeding 100 kb, the reliable PCR amplification of such long genomic regions remains technically challenging due to factors such as GC content, secondary structures, and enzyme processivity. To address this, we chose a PCR-based enrichment strategy using three overlapping primer pairs to amplify ∼10 kb regions across the 25 kb *PCSK9* locus. This approach provides robust, high-fidelity amplification, which is critical for ensuring consistent coverage and accurate variant detection in targeted sequencing applications.

### 2.2 Sample collection and preparation

The Institutional Review Board and the Ethical Committee of Centro Cardiologico Monzino IRCCS approved this study (CCM1068). The investigation conformed to the principles outlined in the Declaration of Helsinki (1964). DNA extraction was conducted from 200 μL of Buffy coat from human blood using QIAamp DNA Mini Kit (QIAGEN), following the manufacturer’s instructions. Samples were eluted in 100 μL AE buffer and quantified using Nanodrop 1,000 (Thermo Fisher Scientific). In order to proceed with the sequencing, the DNA region of interest, a ~ 30 Kbp locus covering all the *PCSK9* gene, was amplified by PCR using the primers indicated in Supplementary Table S1. Briefly, three partially overlapping amplicons (first, second, and third region) of ~10′000 bp, were generated using the conditions described in Supplementary Tables S2, S3. The two non-overlapping amplicons (first and third region) were amplified together, while the amplicon in the second region was generated separately.

During protocol set-up, the amplified DNA was run on a 1% agarose gel to verify that specific amplicons of the desired length were obtained. After that, the two PCR products were pooled, purified using 0.8x volume of AmpPure Beads (Beckman Coulter), and eluted in 35 μL of sterile water.

### 2.3 Library preparation (FLO-MIN106)

The first test run was conducted using a single MinION flow cell FLO-MIN106 R9.4.1 and the ligation sequencing kit (LSK-110), starting from 200 fmol of purified PCR product. We followed the standard protocol and loaded 100 fmol of the library on a MK-1C instrument (Oxford Nanopore Technologies).

After the successful run on a single sample, we proceeded to set up the multiplexing protocol. In order to do that, we extracted total DNA from 12 samples and amplified the *PCSK9* gene locus as described above. All 12 samples were used to prepare the sequencing library following the Native Barcoding amplicons (EXP-NBD104 + SQK-LSK109, Oxford Nanopore Technologies) protocol. In Supplementary Table S4 are described the barcode sequences. In the end, 100 fmol of 12 pooled barcodes were loaded on a MK-1C sequencer using a FLO-MIN106 flow cell (R9.4, Nanopore Technologies).

### 2.4 Library preparation (FLO-FLG001)

After assessing the feasibility of our method using a standard MinION flow cell FLO-FLG001 R9.4.1, we decide to assess whether our protocol could be adapted to a Flongle flow cell. In order to do that, we started from the same DNA used in the multiplexing set-up and followed the same protocol for library preparation up to the adapter ligation and following the clean-up step. All following steps were conducted using the Amplicons by Ligation Protocol (SQK-LSK110) for the Flongle flow cell. In particular, the adapted library, including all 12 barcodes, was purified using 50 μL of AmpPure Beads (Beckman Coulter), incubated for five minutes, and washed with 125 μL of Long Fragment Buffer (LFB) two times. Then, pelleted beads were resuspended in 10 μL of Elution buffer (EB) and incubated for 10 min. In the end, seven microliters of the eluate were used to load the Flongle flow cell following the manufacturer’s protocol.

### 2.5 Basecalling and alignment

All the raw fast5 files obtained from the sequencing run by MinION device were basecalled on Guppy v5.0.15, run on NVIDIA Quadro RTX 5000 GPU, both in high (HAC) and super high (SUP) accuracy mode. These two different basecalling modes differ primarily in terms of accuracy, throughput, and computational requirements. In particular, SUP mode employs a larger, more sophisticated neural network model that increases the accuracy of basecalls at the expense of processing speed. The minimum quality score (Qscore) threshold, used to filter the sequenced reads, is set to 9 and 10 in HAC and SUP mode, respectively. We also filtered out from the analysis the reads with a length lower than 9′500 bp and higher than 11′000 bp, based on our pre-design protocol. Using cutadapt v1.15, the selected reads were demultiplexed based on the ONT barcoded sequences (Supplementary Table S4), which were then trimmed, and mapped to the hg19 human reference genome using Minimap2 v2.22 with optimized parameters (−x map-ont) (15). Each sam file was converted to a sorted and indexed bam format using Samtools v1.13 (16), which was also applied to compute the per-base coverage. Finally, three clusters of reads (bam files) were created per sample based on the mapping PCR regions of the *PCSK9* gene (first: 55′038’604–55′048’959, second: 55′048’029–55′058’118, third: 55′055’369–55′065’748) and were randomly down-sampled to obtain a depth of 50x coverage, i.e., a minimum of 50 unique reads were required for each base of the region considered.

### 2.6 SNVs calling

SNVs were identified from the bam files with a minimum sequencing depth of 50x for the three regions of *PCSK9* for each sample by two different variant callers, Longshot v0.4.1 (9) and PEPPER–Margin–DeepVariant r0.8 (11), specifically designed for long-read sequence data.

To ensure consistency across experiments and isolate the effect of basecalling and platform performance, all sequencing was performed using R9.4.1 flow cells for both MinION and Flongle devices. This uniform chemistry allowed us to attribute differences in yield and read accuracy to basecalling models (HAC vs. SUP) and hardware-specific characteristics, rather than to sequencing chemistry variability.

Longshot was used both for the mapped reads called using HAC and SUP modes, while PEPPER–Margin–DeepVariant pipeline is suitable for SUP mode. Longshot, which uses a haplotype-aware Bayesian model, assigns custom quality scores to each variant, reflecting the log-likelihood ratio between alternative and reference genotypes. To ensure high-confidence variant calls, we applied an empirical quality threshold of >300, derived from an internal quality score distribution analysis across our dataset. This threshold minimized false positives while maintaining a high true-positive rate. In contrast, PEPPER–Margin–DeepVariant leverages DeepVariant’s deep learning-based model for variant scoring and incorporates an internal calibration based on training against benchmark datasets (e.g., GIAB). Therefore, we retained only those variants flagged with FILTER = PASS, in line with the tool developers’ recommendations.

A single annotated VCF file was generated per sample per pipeline, and these were used for downstream accuracy evaluation. For visualization of aligned reads and per-base coverage across the *PCSK9* gene, we used Integrative Genomics Viewer (IGV) v2.11.2 (17).

### 2.7 Statistical modelling

The correlation between the number of long sequenced fragments (10 kbp) to reach at least 50x of depth and samples was evaluated separately for MinION and Flongle sequencing runs. The data points for twelve samples are the real data obtained from our runs, which were then used to infer those for six, three, and one samples. Specifically, assuming additive performance, we pooled the number of fragments obtained from two, four, and all twelve samples to represent the other data points. The best-fitting model to predict the trends increasing the number of sequenced samples was identified. All the data processing steps and plots was carried out using the R software environment v4.1.1.

### 2.8 Sanger sequencing and accuracy evaluation

Sanger sequencing was performed to validate the accuracy of SNV detection obtained by ONT MinION and Flongle flow cell sequencing. We chose three different regions for the validation by Sanger sequencing, and consequently, three pairs of primers were designed (Supplementary Table S5).

The obtained sequences were aligned to the reference genome and the identified SNVs were annotated and compared to those detected through the different proposed workflows. In particular, metrics involving precision, recall, and F1-score for each sample were computed. Finally, a mean F1-score among the samples was associated with each workflow.

## 3 Results

**Figure 1:**
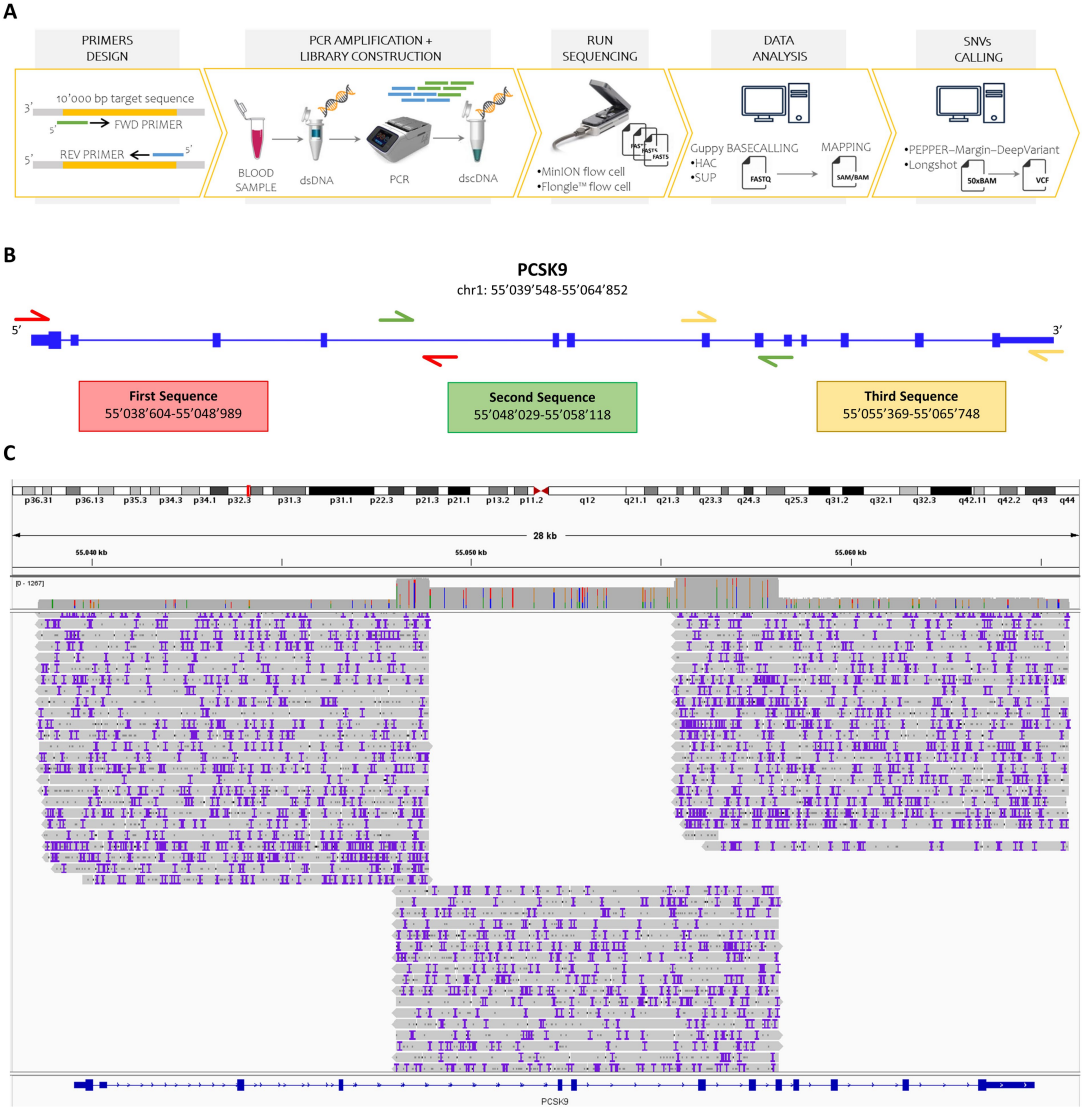
Overview of the study design and sequencing workflow. **(A)** Schematic representation of the nanopore sequencing workflow. Custom forward and reverse primers were designed to target DNA regions up to 10,000 base pairs in length. Double-stranded DNA (dsDNA) was extracted from human blood samples and amplified via polymerase chain reaction (PCR). A barcoded double-stranded cDNA (dscDNA) library was then constructed and loaded onto either a MinION or Flongle flow cell using the SQK-LSK109 kit. Raw signal data (fast5 files) were basecalled using Guppy in both high-accuracy (HAC) and super-accuracy (SUP) modes. The resulting reads were aligned to the human reference genome (GRCh38), and SNVs were identified using PEPPER-Margin-DeepVariant and Longshot, applying a minimum sequencing depth threshold of 50X. **(B)** Primer design strategy. Three overlapping primer pairs were designed to fully cover the *PCSK9* gene, including untranslated regions, ensuring continuous coverage. **(C)** Coverage visualization. Read alignments for the amplified *PCSK9* regions were visualized using Integrative Genomics Viewer (IGV), confirming high coverage and overlap across the targeted loci.

We established a user-friendly workflow based on ONT sequencing to identify homozygous and heterozygous SNVs in the *PCSK9* gene across twelve subjects. The workflow involved: (1) primer design for full gene coverage, (2) PCR amplification, (3) library construction, (4) flow cell loading and sequencing, (5) alignment, and (6) SNV calling (Figure 1A). While the flow cell chemistry (R9.4.1) remained constant, we implemented six variations of the workflow changing several crucial steps, such as different nanopore sequencing flow cells (MinION/Flongle), basecalling models (HAC/SUP), and variant calling algorithms (Longshot/PEPPER).

To amplify the 25’406 bp *PCSK9* locus, three overlapping primer pairs were designed (Supplementary Table S1), including 5′ and 3′ untranslated regions (Figure 1B). For all collected samples, these three DNA fragments were amplified through PCR and a unique barcoded sequencing library was prepared and loaded into MinION and Flongle flow cells.

Following 48-h sequencing runs, raw data underwent basecalling (via Guppy in HAC and SUP modes), filtering, demultiplexing, trimming, and alignment to the reference genome (NC_000001.11).

The sequencing run employing MinION flow cell and basecalling with Guppy in HAC mode resulted in 1′852’058 failed and 7′018’868 passed reads. After the removal of all sequences not spanning the interested region (*PCSK9* locus) or shorter than 9′500 bp, we obtained 1′500’790 reads (Supplementary Figure S1A). Meanwhile, using MinION flow cell and running Guppy in SUP mode, 2′925’381 reads were filtered out and 6′590’059 reads were selected. Of the latter, 1′429’180 reads passed the filtering based on mapping and length (Supplementary Figure S1B). As expected from the claimed performance, Flongle flow cells produced fewer reads regardless of basecalling mode. In particular, 7′587 failed and 243′860 passed reads were obtained running Guppy in HAC mode, while 22′101 failed and 229′346 passed reads with Guppy in SUP mode. Finally, we extracted 40′391 and 38′801 reads, based on the appropriate length, through HAC and SUP basecalling, respectively (Supplementary Figures S1C,D). These selected reads were then demultiplexed, trimmed, and aligned to the reference sequence of the *PCSK9* gene (hg19). More than 98% of the total reads successfully aligned the *PCSK9* sequence gene. Coverage across the entire gene locus was confirmed, with three primary read groups corresponding to each PCR amplicon and additional coverage over overlapping regions (Figure 1C; Supplementary Figure S2).

Aware of the impact of the sequencing depth on the variant calling performance, we aimed to find a balance between cost-effectiveness and data sufficiency. Based on recent studies (18, 19), we set the depth to at least 50x for each base, allowing also the inference of more genomic regions and samples.

Two SNV callers were applied: Longshot (statistical-based) and PEPPER-Margin-DeepVariant (deep learning-based). Longshot was applied on HAC and SUP data, while PEPPER-Margin-DeepVariant was only compatible with SUP mode data.

**Figure 2:**
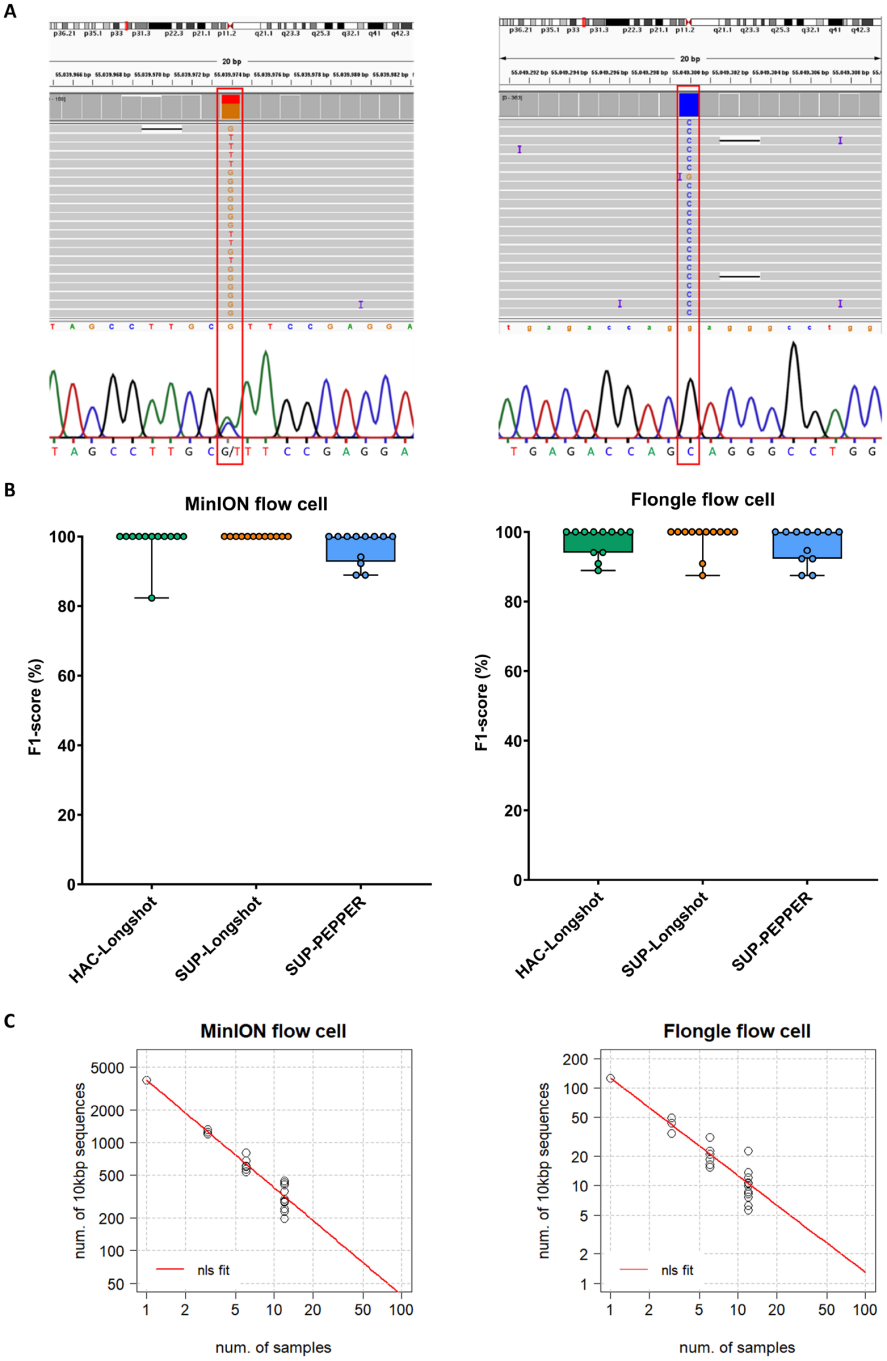
SNV detection performance across MinION and Flongle sequencing workflows. **(A)** Representative examples of heterozygous and homozygous SNVs in the *PCSK9* gene detected by both nanopore sequencing (MinION and Flongle flow cells) and Sanger sequencing. Sanger electropherograms were obtained from the reverse complement strand; reported bases represent the complementary nucleotides. **(B)** F1-score distribution by basecalling mode, variant caller, and flow cell type. Boxplots show the distribution of F1-scores (%) for variant calling pipelines applied to data from MinION (left panel) and Flongle (right panel) flow cells. Each condition combines a basecalling model and variant caller: HAC-Longshot (green): high accuracy basecalling with Longshot variant caller, SUP-Longshot (orange): super accuracy basecalling with Longshot and, SUP-PEPPER (blue): super accuracy basecalling with PEPPER-Margin-DeepVariant caller. Each dot represents an individual barcoded sample. **(C)** Scalability analysis of sequencing throughput. The correlation between the number of 10,000 bp target fragments per sample and the total number of barcoded samples required to achieve a minimum sequencing depth of 50 × is shown for MinION (left) and Flongle (right) flow cells. Black dots represent observed data points from the experimental runs. The red curve represents the predicted values derived from nonlinear least squares (nls) regression fitting.

Among the 9′590 annotated SNVs known for *PCSK9*, a total of 137 unique SNVs were detected across the full-length gene in the twelve evaluated samples (Supplementary Tables S6–S11). Sanger sequencing was performed in targeted regions of *PCSK9* across all samples (21,613 bp total) identified 88 SNVs, serving as a gold standard for performance comparison (Figure 2A; Supplementary Table S13). Based on the resulting true positive (TP), false negative (FN), and false positive (FP) calls, overall performance metrics, including precision, recall, and F1-score, were calculated for each workflow (Figure 2B; Table 1; Supplementary Tables S14–S16).

**Table 1:** Comprehensive performance summary reporting cumulative true positives (TP), false positives (FP), and false negatives (FN) across all samples, alongside the average precision, recall, and F1-scores for each combination of basecalling mode, variant caller, and flow cell.

Basecalling mode	Variant caller	Flow cell	TP	FP	FN	Precision	Recall	F1-score
HAC	Longshot	MinION	85	0	3	100	97.5 ± 8.7	98.5 ± 5.1
SUP	Longshot	MinION	88	0	0	100	100	100
SUP	PEPPER-Margin-DeepVariant	MinION	84	0	4	100	94.5 ± 8.4	97 ± 4.6
HAC	Longshot	Flongle	84	1	4	98.6 ± 4.8	96.5 ± 6.7	97.3 ± 4.1
SUP	Longshot	Flongle	86	1	2	98.6 ± 4.8	98.1 ± 6.4	98.2 ± 4.3
SUP	PEPPER-Margin-DeepVariant	Flongle	81	0	7	100	93.1 ± 9.1	96.2 ± 5.1

Precision, recall, and F1-score data are presented as % mean ± SD.

The MinION-SUP-Longshot workflow achieved perfect concordance with the Sanger reference, reaching 100% precision, recall, and F1-score. MinION-HAC-Longshot and MinION-SUP-PEPPER-Margin-DeepVariant demonstrated slightly lower but consistent performance, with mean F1-scores of 98.5% ± 5.1 and 97.0% ± 4.6, and recall values of 97.5% ± 8.7 and 94.5% ± 8.4, respectively. Notably, no false positives were observed in any MinION-based configuration, resulting in a precision of 100% across all corresponding workflows.

Flongle-based pipelines exhibited slightly greater variability in performance. Flongle-SUP-Longshot achieved the highest accuracy, with a mean F1-score of 98.2% ± 4.3, followed by Flongle-HAC-Longshot (mean F1-score: 97.3% ± 4.1) and Flongle-SUP-PEPPER-Margin-DeepVariant (mean F1-score: 96.2% ± 5.1). Precision remained consistently high across all workflows (≥ 98.6%), although the lowest recall was observed with Flongle-SUP-PEPPER-Margin-DeepVariant (93.1% ± 9.1), indicating a higher incidence of false negatives in some samples.

To further gain insights in the use of MinION and Flongle flow cells in SNV identification, we inferred on the number of genes (long fragments of ⁓10′000 bp) and samples that could be sequenced in one single flow cell. Considering our data with MinION and Flongle flow cells and basecalled by Guppy in SUP mode, we estimated the correlation increasing the samples up to 96 (maximum number of available barcodes) from the nonlinear least-squares (nls) method. Hence, since the data showed a power relationship, a power regression model (y = ax^b^) was fitted in R (Figure 2C). The best-case scenario that could be achieved is 96 samples and ~40 long regions in the MinION flow cell, while in the Flongle flow cell we can infer that ~1 long region could be sequenced for 96 samples simultaneously.

## 4 Discussion

Our findings highlight the potential of nanopore sequencing, particularly using the MinION platform, for accurate SNV detection in long, targeted gene regions. The workflow demonstrated robust performance, especially when combining the SUP basecalling model with the Longshot variant caller, which yielded the most confident variant calls.

To assess the influence of basecalling models on read quality, we maintained a consistent chemistry framework by using R9.4.1 flow cells across all experiments. This controlled setup allowed for a direct comparison between the HAC and SUP basecalling models. Our results showed that the SUP model consistently provided higher per-read accuracy, leading to improved variant confidence and a notable reduction in false positive calls. Although both the MinION and Flongle platforms employed the same sequencing chemistry, we observed that MinION produced substantially higher read yields and more uniform coverage. This directly translates into enhanced sensitivity for SNV detection, particularly in regions of lower complexity or coverage depth. In contrast, the Flongle flow cell, while more cost-effective, exhibited reduced throughput and slightly diminished variant detection performance. Therefore, at present, it may be more suitable for pilot studies rather than high-throughput applications requiring high-confidence data.

Beyond basecalling models, our benchmarking results also highlight important performance differences between the two variant calling algorithms evaluated. Longshot, which applies a haplotype-aware Bayesian approach, achieved superior sensitivity across both MinION and Flongle datasets, and was the only method to reach 100% concordance with Sanger sequencing when paired with SUP basecalling. In contrast, PEPPER-Margin-DeepVariant, which uses a deep learning framework trained on whole-genome reference datasets, showed a higher rate of false negatives, particularly in our targeted long-amplicon setting. This reduced sensitivity may reflect conservative filtering thresholds tuned to avoid false positives in genome-wide contexts, or challenges in resolving local sequence complexity within limited read clusters. While PEPPER-Margin-DeepVariant remains a valuable tool for large-scale analyses and has shown high overall performance in whole-genome studies, our results suggest that Longshot may offer greater reliability for variant detection in targeted long-read panels, where maximizing sensitivity without compromising precision is essential. This distinction is particularly relevant in clinical applications where missing pathogenic variants could impact diagnostic outcomes.

In literature, Genome in a Bottle (GIAB) benchmarks (20, 21) and data from Nanopore WGS Consortium (22) have been extensively exploited for studies on the accuracy of variant calls resulting from the total human genome (7, 11, 12). Indeed, these Consortiums offer sequencing data generated by multiple technologies, including ONT platform, to generate variant calls and region. In this context, high levels of accuracy on nanopore-based SNV calls were achieved at whole-genome level both by Longshot (F1-score of 97.7%) and PEPPER-Margin-DeepVariant (F1-score of 99.7%). Our data show that this is also true for targeted long gene sequences, but only for the MinION flow cell, leaving room for improvement for sequencing with Flongle flow cell.

Scalability modelling further supports the feasibility of multiplexing up to 96 samples with ~40 long fragments each using the MinION platform. However, these projections are based on an idealized model derived from extrapolated data obtained from a limited experimental setup. This model assumes uniform read distribution, efficient demultiplexing, and consistent amplification success, which may not fully reflect the variability encountered in real-world sequencing runs. In practice, increasing the number of barcodes can lead to barcode misassignment and reduced per-sample sequencing depth, potentially impacting SNV detection accuracy. Therefore, while our results demonstrate strong potential for scaling the workflow, further experimental validation is necessary to evaluate performance under high-multiplexing conditions. Although our inference is based on extrapolated data from three long fragments across twelve subjects, the results suggest strong potential for expanding this workflow in clinical or translational research.

Our study also points toward applications in diagnostic panels for oncology and cardiology, where multi-gene profiling is essential. The long-read approach can improve variant calling in challenging genomic regions and reduce the need for complex assembly or multiple short-read amplicons. While our workflow was shown using the *PCSK9* gene as a model, the methodology is highly adaptable to other clinically relevant genes. For instance, in cardiology, genes such as LDLR, APOB, SCN5A, MYH7, and TNNT2 are frequently implicated in conditions like familial hypercholesterolemia, arrhythmias, and cardiomyopathies, many of which harbour variants in regions that are difficult to resolve using short-read technologies. In oncology, particularly in breast cancer (mammary carcinoma), long-read sequencing can enhance the detection of variants in genes such as BRCA1, BRCA2, TP53, PIK3CA, and CHEK2, which are critical for risk stratification, therapeutic targeting, and treatment response monitoring.

The use of Oxford Nanopore sequencing allows for flexible amplicon design and the ability to target longer genomic regions in a single read, reducing coverage gaps and enabling accurate phasing of variants. Given the growing clinical use of nanopore sequencing (18, 23) our approach is well-suited for scalable implementation across diverse gene panels. It supports multiplexing and high-throughput workflows, facilitating integration into automated pipelines for routine clinical diagnostics. However, before reaching the clinical stage, the gene panels must be experimentally verified. Thus, in the near future, this strategy holds strong potential for widespread application in precision medicine, enabling rapid and cost-effective detection of variants in a range of clinically significant genes in both oncology and cardiovascular genomics.

However, several limitations must be acknowledged. Our study has a modest sample size and lacks validation against publicly available datasets. While we aimed to establish a controlled, proof-of-concept workflow using clinically derived samples and orthogonal validation by Sanger sequencing, future studies will be required to assess generalizability and reproducibility across broader datasets and sequencing contexts. The focus on a single gene with moderately high GC content and the absence of structural variant analysis may limit generalizability. Furthermore, while our modelling indicates high scalability, further experimental validation is necessary.

Overall, our findings underscore the importance of jointly optimizing sequencing hardware, basecalling strategies, and variant calling algorithms to ensure accurate and reproducible SNV identification in both clinical and research contexts. This study provides foundational evidence supporting the use of ONT-based workflows for high-accuracy SNV detection across long genomic regions, highlighting their clear translational potential in precision medicine and genomic diagnostics.

## 5 Conclusion

We present a successful nanopore-based workflow to identify homozygous and heterozygous SNVs in the *PCSK9* gene. The workflow, which includes steps from primer design to variant calling, was tested varying flow cell types, basecalling modes, and variant caller methods. The best SNV calling performance was achieved by the sequencing run in MinION flow cell, combining the SUP basecalling mode and Longshot variant caller (MinION-SUP-Longshot workflow). On the other hand, Flongle flow cell remains a cost-effective option but with low accuracy. These findings highlight the potential of nanopore sequencing for targeted gene analysis, although further optimization is needed for broader application.

## Data Availability

The raw sequencing data generated in this study are available in the NCBI SRA under BioProject accession number PRJNA1255874. The data can be accessed at: https://www.ncbi.nlm.nih.gov/bioproject/PRJNA1255874.

## Footnotes

^1^ https://primalscheme.com
