# Supplementary material for: Accurate and rapid single nucleotide variation detection in PCSK9 gene using nanopore sequencing
Source: Front Med (Lausanne). 2025 Aug 26;12:1620405. doi: 10.3389/fmed.2025.1620405 (PMC12417156; doi:10.3389/fmed.2025.1620405)
Supplement: Supplementary file 1 [file Data_Sheet_1.pdf]

Supplementary Material

Supplementary Figures

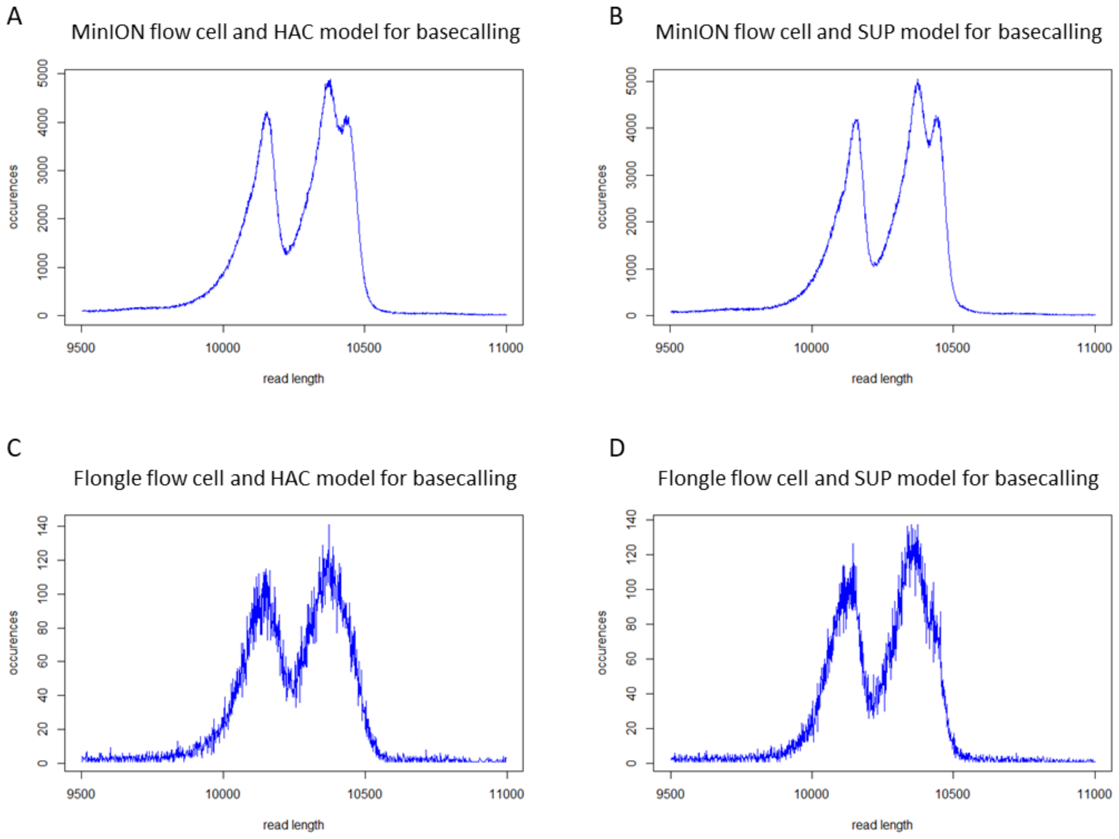

Figure S1. Length distribution of total passed reads spanned 9’500-11’000 bp. **(A)** Length distribution of reads sequenced by MinION flow cell and basecalled by Guppy in HAC mode. **(B)** Length distribution of reads sequenced by MinION flow cell and basecalled by Guppy in SUP mode. **(C)** Length distribution of reads sequenced by Flongle flow cell and basecalled by Guppy in HAC mode. **(D)** Length distribution of reads sequenced by Flongle flow cell and basecalled by Guppy in SUP mode.

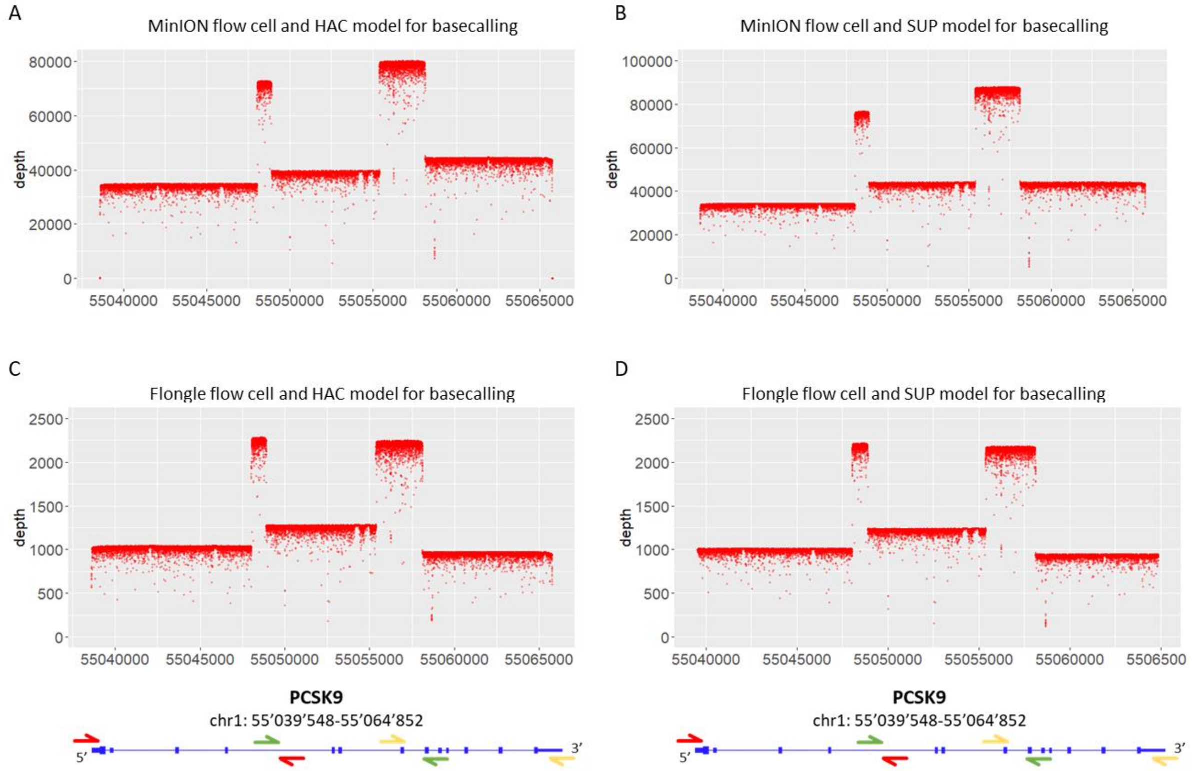

Figure S2. Average coverage distribution. **(A)** Average distribution of reached coverage from sequencing by MinION flow cell and basecalling by Guppy in HAC mode and **(B)** from sequencing by MinION flow cell and basecalling by Guppy in SUP mode. **(C)** Average distribution of reached coverage from sequencing by Flongle flow cell and basecalling by Guppy in HAC mode and **(D)** from sequencing by Flongle flow cell and basecalling by Guppy in SUP mode.

Supplementary Tables

Table S1. PCR primers for PCSK9 PCR amplification.

Title PCSK9 regions	*Direction*	*Sequence (5′ – 3′)*
First Region	FWD	GGAGTGAGGTCTTTGCAAAC
(55′038′604 - 55′048′989)	REV	AAATGCAGGGCTAAAATCAC
Second Region	FWD	GTT TCC TAG CAC TGC TGT GAC A
(55′048′029 – 55′058′118)	REV	TGG CAG AGA AGT GGA TCA GTC T
Third Region	FWD	TCA TCC TCT TGG TGC CTC TCT T
(55′055′369 – 55′065′748)	REV	TTC AAG CCA TTG CCA TCT GTC A

Table S2. PCR conditions to amplify the entire PCSK9 gene.

Compound	***Volume [µl]***
LongAmp Taq 2X Master Mix (NEB)	25
10 µM first Primer mix (1 or 2)	3
10 µM each Primer mix 3 or H2O	3
10 mM dNTPs (NEB)	1
Template DNA (90ng/µl)	3
Nuclease-free water	15
FINAL VOLUME	50

Table S3. PCR thermal protocol.

Step	*Temp*	*Time*
Initial Denaturation	95°C	30 seconds
40 cycles	95°C	10 seconds
	55°C	30 seconds
	65°C	15 minutes
Final Extension	65°C	10 minutes
Hold	4°C	

Table S4. Sequences of Oxford Nanopore Technologies barcodes.

ID	*Sequence*
BC01	AAGAAAGTTGTCGGTGTCTTTGTG
BC02	TCGATTCCGTTTGTAGTCGTCTGT
BC03	GAGTCTTGTGTCCCAGTTACCAGG
BC04	TTCGGATTCTATCGTGTTTCCCTA
BC05	CTTGTCCAGGGTTTGTGTAACCTT
BC06	TTCTCGCAAAGGCAGAAAGTAGTC
BC07	GTGTTACCGTGGGAATGAATCCTT
BC08	TTCAGGGAACAAACCAAGTTACGT
BC09	AACTAGGCACAGCGAGTCTTGGTT
BC10	AAGCGTTGAAACCTTTGTCCTCTC
BC11	GTTTCATCTATCGGAGGGAATGGA
BC12	CAGGTAGAAAGAAGCAGAATCGGA

Table S5. PCR primers for Sanger sequencing validation.

Sanger Sequencing	***Directi on***	***Sequence (5' – 3')***
55'039'574 – 55'040'375	FWD	AGGCGGGCGCCGCCGTTCAGTTC
	REV	ATCTGGGCAGGATATCCTGGCAG
55'048'180 – 55'048'869	FWD	CCCAACAGCGGCAGCACACCAATC
	REV	TCAATACCTGACAGAGAAATGCATGC
55'048'995 – 55'049'691	FWD	GTGGTCTCTTCCCCCAACAGTGTATC
	REV	GTAAAAGCCCCCACAGGAACATCC

Supplementary Methods

Bash script for end-to-end analysis of ONT MinION sequencing data targeting the PCSK9 gene.

This script provides the key steps implemented for the end-to-end analysis of ONT sequencing data targeting the PCSK9 gene. It includes modules for:

- Basecalling using Guppy (HAC and SUP models)
- Length-based read filtering (9.5–11 kb range)
- Barcode demultiplexing using cutadapt
- Alignment to the human genome (hg19) with minimap2
- Coverage analysis using samtools
- Targeted downsampling to ~50× in PCSK9 regions
- Variant calling with PEPPER-Margin-DeepVariant (via Docker) and Longshot

A Linux environment with GPU support is required for running this workflow. All file paths, environment variables, and parameters should be customized to match the user's local system configuration and experimental design.

```
#!/bin/bash
set -euo pipefail

# ========== USER CONFIGURATION ==========

# Base input/output paths (update these)
FAST5_DIR="/path/to/raw_fast5"
BASECALL_HAC_DIR="/path/to/output/fastq_HAC"
BASECALL_SUP_DIR="/path/to/output/fastq_SUP"
REF_HG19="/path/to/reference/hg19.fa"
REF_GRCh38="/path/to/reference/Homo_sapiens.GRCh38.dna.fa"

THREADS=32
BARCODES=12
MIN_LEN=9500
MAX_LEN=11000
TARGET_COVERAGE=50

# Regions of PCSK9 gene in hg19 coordinates
PCSK9_REGIONS=(
  "chr1:55038604-55048959"
  "chr1:55048029-55058118"
  "chr1:55055369-55065748"
)

# Config files for Guppy
```

```
CONFIG_HAC="dna_r9.4.1_450bps_hac.cfg"
CONFIG_SUP="dna_r9.4.1_450bps_sup.cfg"

# ========== STEP 1: Basecalling ==========

echo "Basecalling with Guppy HAC and SUP..."
guppy_basecaller -i "$FAST5_DIR" -s "$BASECALL_HAC_DIR" -c
"$CONFIG_HAC" --device cuda:0 -q 9 --compress_fastq
guppy_basecaller -i "$FAST5_DIR" -s "$BASECALL_SUP_DIR" -c
"$CONFIG_SUP" --device cuda:0 -q 10 --compress_fastq

# ========== STEP 2: Merge and decompress ==========

echo "Decompressing and merging FASTQ..."
for BASECALL in "$BASECALL_HAC_DIR" "$BASECALL_SUP_DIR"; do
  cd "$BASECALL"
  gzip -d -f *.gz
  cat *.fastq > total.fastq
done

# ========== STEP 3: Length filtering ==========

filter_reads_by_length() {
  local input_fastq=$1
  local output_fastq=$2

  awk -v min=$MIN_LEN -v max=$MAX_LEN 'BEGIN {OFS = "\n"}
  {
    h=$0; getline s; getline qh; getline qs;
    if (length(s) >= min && length(s) <= max) print h, s, qh, qs
  }' "$input_fastq" > "$output_fastq"
}

echo "Filtering reads by length ($MIN_LEN - $MAX_LEN bp)..."
filter_reads_by_length "$BASECALL_HAC_DIR/total.fastq"
"$BASECALL_HAC_DIR/filtered.fastq"
filter_reads_by_length "$BASECALL_SUP_DIR/total.fastq"
"$BASECALL_SUP_DIR/filtered.fastq"

# ========== STEP 4: Demultiplexing with cutadapt ==========

demultiplex_barcodes() {
  local fastq=$1
  local output_dir=$2
  mkdir -p "$output_dir"
  cd "$output_dir"

  echo "Running forward and reverse barcode matching..."
```

```
  cutadapt -e 0.2 \
    -a s1=AAGAAAGTTGTCGGTGTCTTTGTG -a s2=TCGATTCCGTTTGTAGTCGTCTGT \
    -a s3=GAGTCTTGTGTCCCAGTTACCAGG -a s4=TTCGGATTCTATCGTGTTTCCCTA \
    -a s5=CTTGTCCAGGGTTTGTGTAACCTT -a s6=TTCTCGCAAAGGCAGAAAGTAGTC \
    -a s7=GTGTTACCGTGGGAATGAATCCTT -a s8=TTCAGGGAACAAACCAAGTTACGT \
    -a s9=AACTAGGCACAGCGAGTCTTGGTT -a s10=AAGCGTTGAAACCTTTGTCCTCTC
\
    -a s11=GTTTCATCTATCGGAGGGAATGGA -a s12=CAGGTAGAAAGAAGCAGAATCGGA
\
    -o unknown.fwd.fastq "$fastq" --no-trim --quiet

  cutadapt -e 0.2 \
    -a s1=CACAAAGACACCGACAACTTTCTT -a s2=ACAGACGACTACAAACGGAATCGA \
    -a s3=CCTGGTAACTGGGACACAAGACTC -a s4=TAGGGAAACACGATAGAATCCGAA \
    -a s5=AAGGTTACACAAACCCTGGACAAG -a s6=GACTACTTTCTGCCTTTGCGAGAA \
    -a s7=AAGGATTCATTCCCACGGTAACAC -a s8=ACGTAACTTGGTTTGTTCCCTGAA \
    -a s9=AACCAAGACTCGCTGTGCCTAGTT -a s10=GAGAGGACAAAGGTTTCAACGCTT
\
    -a s11=TCCATTCCCTCCGATAGATGAAAC -a s12=TCCGATTCTGCTTCTTTCTACCTG
\
    -o unknown.rev.fastq unknown.fwd.fastq --no-trim --quiet

  for i in $(seq 1 $BARCODES); do
    cat s${i}.fwd_barcode.fastq s${i}.rev_barcode.fastq >
BRC${i}.fastq
  done
}

echo "Demultiplexing HAC filtered reads..."
demultiplex_barcodes "$BASECALL_HAC_DIR/filtered.fastq"
"./demux_HAC"

# ========== STEP 5: Alignment with minimap2 ==========

echo "Running alignment with minimap2..."
for i in $(seq 1 $BARCODES); do
  minimap2 -ax map-ont "$REF_HG19" "demux_HAC/BRC${i}.fastq" >
"demux_HAC/BRC${i}.sam"
done

# ========== STEP 6: Convert, sort, and index BAM ==========

echo "Converting SAM to sorted/indexed BAM..."
for i in $(seq 1 $BARCODES); do
  samtools view -bS "demux_HAC/BRC${i}.sam" | samtools sort -o
"demux_HAC/BRC${i}.sorted.bam"
  samtools index "demux_HAC/BRC${i}.sorted.bam"
done
```

```
# ========== STEP 7: Coverage analysis ==========

echo "Generating coverage reports..."
for i in $(seq 1 $BARCODES); do
  samtools depth "demux_HAC/BRC${i}.sorted.bam" >
"demux_HAC/BRC${i}.coverage.txt"
done

# ========== STEP 8: Downsampling ==========

echo "Downsampling PCSK9 gene regions to ~${TARGET_COVERAGE}x..."
for i in $(seq 1 $BARCODES); do
  for region in "${PCSK9_REGIONS[@]}"; do
    region_tag=$(echo "$region" | tr ':' '-' | tr '-' '_')
    bam_in="demux_HAC/BRC${i}.sorted.bam"
    bam_out="demux_HAC/BRC${i}_${region_tag}_50x.bam"

    samtools view -b "$bam_in" "$region" > temp.bam

    # Estimate and apply downsampling factor manually if needed
    samtools view -s 0.1 -b temp.bam > "$bam_out"
    samtools index "$bam_out"
    rm temp.bam
  done
done

# ========== STEP 9: Variant Calling ==========

run_variant_calling() {
  local input_bam=$1
  local output_dir=$2
  local prefix=$3

  echo "Calling variants with PEPPER-DeepVariant..."
  sudo docker run -v "$PWD":"$PWD" kishwars/pepper_deepvariant:r0.8
\
    run_pepper_margin_deepvariant call_variant \
    -b "$input_bam" \
    -f "$REF_GRCh38" \
    -o "$output_dir" \
    -p "$prefix" \
    -t "$THREADS" \
    --ont_r9_guppy5_sup

  echo "Calling variants with Longshot..."
  samtools sort -@ "$THREADS" -o "${input_bam%.bam}.sorted.bam"
"$input_bam"
  samtools index "${input_bam%.bam}.sorted.bam"
```

```
  longshot \
    --bam "${input_bam%.bam}.sorted.bam" \
    --ref "$REF_GRCh38" \
    --out "${output_dir}/${prefix}_longshot.vcf" \
    --num_threads "$THREADS"
}

run_variant_calling "demux_HAC/BRC1_chr1_55038604_55048959_50x.bam"
"./variants_BRC1" "BRC1"

echo "Pipeline completed successfully."
```
